# An Intriguing Shift Occurs in the Novel Protein Phosphatase 1 Binding Partner, TCTEX1D4: Evidence of Positive Selection in a Pika Model

**DOI:** 10.1371/journal.pone.0077236

**Published:** 2013-10-10

**Authors:** Luís Korrodi-Gregório, Ana Margarida Lopes, Sara L. C. Esteves, Sandra Afonso, Ana Lemos de Matos, Andrey A. Lissovsky, Odete A. B. da Cruz e Silva, Edgar F. da Cruz e Silva, Pedro José Esteves, Margarida Fardilha

**Affiliations:** 1 Laboratory of Signal Transduction, Centre for Cell Biology, Health Sciences Department and Biology Department, University of Aveiro, Aveiro, Portugal; 2 CIBIO/UP, Centro de Investigação em Biodiversidade e Recursos Genéticos/Universidade do Porto, InBio, Laboratório Associado, Vairão, Portugal; 3 Departamento de Zoologia e Antropologia, Faculdade de Ciências da Universidade do Porto, Porto, Portugal; 4 INSERM, Institut National de la Santé et de la Recherche Médicale, Unit 892, Université de Nantes, Nantes, France; 5 Department of Microbiology and Immunology, Stritch School of Medicine, Loyola University Chicago, Maywood, Illinois, United States of America; 6 Zoological Museum, Moscow State University, Moscow, Russia; 7 Laboratory of Neurosciences, Centre for Cell Biology, Health Sciences Department and Biology Department, University of Aveiro, Aveiro, Portugal; 8 CESPU, Instituto de Investigação e Formação Avançada em Ciências e Tecnologias da Saúde, Gandra PRD, Portugal; Emory University, United States of America

## Abstract

T-complex testis expressed protein 1 domain containing 4 (TCTEX1D4) contains the canonical phosphoprotein phosphatase 1 (PPP1) binding motif, composed by the amino acid sequence RVSF. We identified and validated the binding of TCTEX1D4 to PPP1 and demonstrated that indeed this protein is a novel PPP1 interacting protein. Analyses of twenty-one mammalian species available in public databases and seven Lagomorpha sequences obtained in this work showed that the PPP1 binding motif _90_RVSF_93_ is present in all of them and is flanked by a palindromic sequence, PLGS, except in three species of pikas (*Ochotona princeps*, *O. dauurica* and *O. pusilla*). Furthermore, for the *Ochotona* species an extra glycosylation site, motif _96_NLS_98_, and the loss of the palindromic sequence were observed. Comparison with other lagomorphs suggests that this event happened before the *Ochotona* radiation. The d_N_/d_S_ for the sequence region comprising the PPP1 binding motif and the flanking palindrome highly supports the hypothesis that for *Ochotona* species this region has been evolving under positive selection. In addition, mutational screening shows that the ability of pikas TCTEX1D4 to bind to PPP1 is maintained, although the PPP1 binding motif is disrupted, and the N- and C-terminal surrounding residues are also abrogated. These observations suggest pika as an ideal model to study novel PPP1 complexes regulatory mechanisms.

## Introduction

Phosphoprotein phosphatase 1 (PPP1), one of the major eukaryotic serine/threonine protein phosphatases, has exquisite specificities *in vivo*, both in terms of substrates and cellular localization. Over the past two decades, it has become apparent that PPP1 versatility is achieved by its ability to interact with multiple targeting/regulatory subunits known as PPP1 interacting proteins [1,2]. To date, more than 200 interacting proteins have been identified, most of them having the consensus PPP1 binding motif (RVxF), that binds to the catalytic subunit of PPP1 (PPP1C), determining its targeting and thus specifying cellular location and ultimately function [3,4]. The RVxF motif is present in about 70% of all PPP1 interacting proteins [3]. This motif is usually surrounded by basic residues in the N-terminal and by acidic residues in the C-terminal. The binding of this motif to a hydrophobic groove in PPP1C does not alter PPP1C conformation, but anchors the interacting proteins to PPP1C [5-8]. Nevertheless, the initial binding of this motif to PPP1C is essential to bring the PPP1 interacting proteins into its proximity, allowing for secondary interactions that strength holoenzyme binding, determining substrate specificity, enzyme activity and PPP1 isoform selectivity [9]. Therefore, the key to characterize the diverse roles of PPP1 is the identification of novel interacting proteins and understand the PPP1 complexes specific functions. Thus, several novel PPP1 interacting proteins have been identified, through a yeast two-hybrid system, using PPP1 as bait [10-14].

A novel partner of PPP1 was identified recently and described as a novel Tctex1 dynein light chain family member, the t-complex testis expressed protein 1 domain containing 4, TCTEX1D4 (Tctex2β) [14,15]. Cytoplasmic dyneins are protein complexes responsible for the retrograde transport, minus-end directed trafficking in the cytoskeletal microtubules [16]. More specifically, the light chains can confer specificity to cargo binding [17,18], regulate other molecules [19] or stabilize the assembly of the motor dynein complex [20]. It was already shown that TCTEX1D4 interacts with membrane receptors, inhibiting TGFβ signaling [15] and suggesting its involvement in brain response to peripheral inflammation [21]. Previous results indicate that TCTEX1D4 is evolutionarily conserved among mammals and ubiquitously expressed, particularly in ovary, spleen, lung and placenta, where PPP1 is also present [22,23]. Moreover TCTEX1D4 interacts directly with PPP1C [22] and possesses a canonical PPP1 binding motif [5,8,24,25]. We have also demonstrated that TCTEX1D4 and PPP1C co-localize in the microtubule organizing center and in microtubules having a probable role in the cytoplasmic transport of the cell [22].

The TCTEX1D4 PPP1 binding motif, _90_RVSF_93_ (amino acid 90 to 93, according to *Homo sapiens* sequence), was shown to be present in all mammals except in one lagomorpha species, *Ochotona princeps* (subgenus Pika) [22]. The order Lagomorpha is divided into the families Ochotonidae (pikas) and Leporidae (rabbits and hares). Ochotonidae has a single genus (*Ochotona* sp.), which is divided in three subgenera (Conothoa, *Ochotona* and Pika) [26]. On the other hand, Leporidae encompasses eleven genera [27,28], which includes *Oryctolagus*, *Lepus* and *Sylvilagus* genera that diverged at 12 million years ago (mya) [29-33]. In spite of the studies performed in the past few years, using fossil and molecular data, the divergence time between these two families remains vague. According to Matthee et al. [29], the leporid-ochotonid split was around 31mya. McKenna and Bell [34] and Asher et al. [35] suggested that the families’ separation occurred around 37 my ago. Three other authors [36-38] suggested 65 my as the leporid-ochotonid divergence time. These different molecular dating models of leporid-ochotonid separation were used by Lanier and Olson [39] to infer a common ancestor for pikas. However, the *Ochotona* genus taxonomy is still poorly resolved [39-42]. Lanier and Olson [39] suggested radiation time estimations for the *Ochotona* subgenera Pika (between 6 and 13 mya), Conothoa (between 7 and 16 mya) and *Ochotona* (between 10 and 20 mya).

In this work we compared different lagomorphs, *Oryctolagus*, *Lepus*, *Sylvilagus* and *Ochotona* TCTEX1D4. Our main goal was to validate the observation that TCTEX1D4 PPP1 binding motif is absent across *Ochotona* species and to evaluate the evolution of this protein in the Lagomorphs. Also, different mutants mimicking Pika PPP1 binding motif and surrounding amino acids were produced and the binding efficiency was determined by the overlay technique. These findings were applied to understand the evolutionary mechanisms that are behind these dramatic amino acid changes.

## Materials and Methods

### Samples and ethics statement

*Ochotona* samples were loaned by the Zoological Museum of Moscow State University, Moscow, Russia, under the supervision of the Lagomorph museum curator Andrey A Lissovsky. Genomic DNA samples of *Lepus americanus* and *Sylvilagus floridanus* were provided by the Department of Microbiology and Immunology of Loyola University Chicago, Illinois, USA. *Lepus europaeus* and *Lepus granatensis* tissue samples were supplied by CIBIO, Vairão, Portugal and *Sylvilagus bachmani* tissue samples were provided by the Blue Oak Ranch Reserve of the University of California, California, USA. It was not necessary to obtain approval from an ethics committee for *Lepus* and *Sylvilagus* samples because these samples were already described and used in previous publications [30,31,43-45].

### Analyses of TCTEX1D4 evolution

Twenty-one different mammal protein sequences from TCTEX1D4 were collected from NCBI GenBank (http://www.ncbi.nlm.nih.gov/) and from Ensembl (http://www.ensembl.org/index.html/). Table S1 contains the denominations, GenBank accession numbers and Ensembl scaffolds for all the acquired sequences. Additionally, two other TCTEX1D4 protein sequences from *Ochotona* species, three sequences from *Lepus* species and two from *Sylvilagus* species were sequenced in this study.

Tissue samples from *Ochotona dauurica* (Ocda) and *Ochotona pusilla* (Ocpu), belonging to the subgenus *Ochotona*, were used. Genomic DNA was extracted using the E.Z.N.A. ^®^ Tissue DNA Kit (Omega Bio-Tek, Norcross, Georgia, USA) according to manufacturer’s instructions. A pair of primers was designed according to the sequence for *Ochotona princeps* (Ocpr) in Ensembl (forward 5’-ATGGCTGGCAGGCCTCTGCC-3’ and reverse 5’-CTCGCAGTAGAGCCCGTGGA-3’) generating a PCR fragment of 657bp. A touchdown PCR was performed and the thermal profile used was the following: initial denaturation (95°C for 15min.); 5 cycles of denaturation (95°C for 30sec.), annealing (66°C for 30sec., 1°C decrease/cycle) and extension (72°C for 45sec.); 30 cycles of denaturation (95°C for 30sec.), annealing (62°C for 30sec.) and extension (72°C for 45sec.); and a final extension (72°C for 20min.). Sequencing was performed on an ABI PRISM 310 Genetic Analyzer (Perkin-Elmer, Applied Biosystems, Barcelona, Spain), where the ABI PRISM BigDye Terminator Cycle sequencing protocols were followed.

Total RNA isolation and cDNA synthesis (guanidinium thiocyanate-phenol-chloroform extraction) were performed in tissues of *Lepus europaeus* (Leeu), *Lepus granatensis* (Legr) and *Sylvilagus bachmani* (Syba). Genomic DNA samples of *Lepus americanus* (Leam) and *Sylvilagus floridanus* (Syfl) were also used. A set of primers was designed according to the available sequence for *Oryctolagus cuniculus* (Orcu) existent in Ensembl (forward 5’ TGCCAGGAGGAGGAGACTG 3’ and reverse 5’ CACGCTGCACACCAGCTTG 3’) generating a PCR fragment of approximately 500bp. The PCR thermal profile used was the following: initial denaturation (95°C for 3min.); 40 cycles of denaturation (95°C for 45sec.), annealing (57°C for 1min.) and extension (72°C for 1min.); and a final extension (72°C for 10min.).

The nucleotide sequences were translated and aligned using ClustalW [46] and adjusted by visual examination (data not shown). The sequences obtained in this work have been deposited into NCBI GenBank under the accession numbers: KF360247-253 (7 sequences). Maximum Likelihood phylogenetic reconstruction was performed for the whole TCTEX1D4 gene alignment and for the specific twelve amino acid region (four upstream and four downstream of the motif _90_RVSF_93_). As indicated by the Akaike information criterion (AIC) implemented in jModelTest v0.1.1 [47], the nucleotide substitution model TVM+G was used for the whole gene tree estimation, while the TIM2+I+G model was selected as the best-fit nucleotide substitution model for the twelve amino acid region. For the Maximum Likelihood phylogenetic analyses we used GARLI v2.0 (Genetic Algorithm for Rapid Likelihood Inference) [48] applying 1,000,000 generations and 1,000 bootstrap searches. Maximum Likelihood trees were displayed using FigTree v1.3.1 (http://tree.bio.ed.ac.uk/).

### Signature of selection and sliding-window analysis

Under neutrality, the expected ratio of non-synonymous (d_N_) to synonymous (d_S_) substitutions in a gene is one (Ka/Ks= d_N_/d_S_=ω=1) and significant deviations from this value can be interpreted as evidence of either positive selection (ω>>1) or purifying/negative selection (ω<<1). To consider a specific pattern of nucleotide substitution, synonymous and non-synonymous substitution rates were estimated using the Nei-Gojobori method [49] and ω was calculated. To determine the nucleotide substitution rate variation among different nucleotide regions we can plot the differences as averages by sliding a window along a sequence alignment [50]. A 234 nucleotide region (between nucleotides 151 and 384 of the codifying sequence of *Ochotona princeps*) encompassing the palindromic region (between nucleotides 250 and 285) was selected and the alignment was performed using the software package MEGA 4.1 [51]. The sliding-window analysis was performed using DnaSP version 5.10 [52]. A window length of 9 nucleotides and a step size of 3 were chosen for this analysis. The ratio of non-synonymous to synonymous substitutions between Rabbit/Pika, Rabbit/Mouse and Rabbit/Rat was then analyzed. Final plots were obtained using SigmaPlot (SigmaPlot v.11, Systat Software, San Jose, California, USA).

### Site-direct mutagenesis

Mutagenic primers were designed according to the sequence of human TCTEX1D4 (NCBI: NM_001013632.2) and used to obtain the desired mutations (Table 1). Starting with pET-TCTEX1D4 plasmid as template, and along with appropriate mutagenic primers, the mutants HA+INL+WS, HA+WS, HA+INL, INL+WS, HA, INL and WS were created using the QuikChange^®^ Site-Directed Mutagenesis Kit (Stratagene, Agilent Technologies UK Ltd, Edinburgh, UK). PCR conditions for site-directed mutagenesis were as followed: initial denaturation (95°C for 1min.); 18 cycles of denaturation (95°C for 30sec.), annealing (55°C for 1min.) and extension (68°C for 7min.), using KOD polymerase (Novagen, Madison, Wisconsin, USA). DNA was then digested by DpnI restriction enzyme and transformed into *E. coli* XL1-Blue strain (Stratagene Agilent Technologies UK Ltd, Edinburgh, UK). Sequencing was performed on an ABI PRISM 310 Genetic Analyzer (Perkin-Elmer, Applied Biosystems, Barcelona, Spain), where the ABI PRISM BigDye Terminator Cycle sequencing protocols were followed. Positive clones were sequenced using universal T7 promoter and T7 terminator primers.

**Table 1:** TCTEX1D4 mutant constructs.

*Homo sapiens* (Humans)			*Ochotona princeps* (Pika)
_85_ **PP**LGSR**VSF**SG**LP**_97_			_83_ **HA**LGSR**INL**SG**WS**_95_
**Mutation**	**Primer Name**	**Sequence**	
_85_PP_86_ to _85_HA_86_	HA-FW	5’-GGGCCCGGTGCACGCTCTGGGCTCAAG-3’	
	HA-RV	5’-CTTGAGCCCAGAGCGTGCACCGGGCCC-3’	
_91_VSF_93_ to _91_INL_93_	INL-FW	5’-CTCTGGGCTCAAGGATCAACTTATCAGGGTTGCCCC-3’	
	INL-RV	5’-GGGGCAACCCTGATAAGTTGATCCTTGAGCCCAGAG-3’	
_97_LP_98_ to _97_WS_98_	WS-FW	5’-GCTTCTCAGGGTGGTCCCTGGCGCCCG-3’	
	WS-RV	5’-CGGGCGCCAGGGACCACCCTGAGAAGC-3’	
_97_LP_98_ to _97_WS_98_	L..WS-FW	5’-CAACTTATCAGGGTGGTCCCTGGCGCCCGCC-3’	
	L..WS-RV	5’-GGCGGGCGCCAGGGACCACCCTGATAAGTTG-3’	

Human and Pika PPP1 binding motif and surrounding sequences are shown on the top. *Mutation sites are highlighted in bold and underlined. On the bottom, oligonucleotides used for site direct mutagenesis are shown.*

### Protein expression and overlay assay

Each His-tagged mutant was transformed into *E. coli* Rosetta strain (Novagen, Madison, Wisconsin, USA). A single colony was selected and grown overnight at 37°C in the appropriate media until an optical density of 0.6-0.7 was reached. Expression was induced using 1M IPTG (isopropyl-β-D-thio-galactopyranoside), at 37°C with shaking, for 3hrs. Culture cells were recovered by centrifugation and treated as described elsewhere [12]. Lysates were then mass normalized using a BCA^®^ assay (Fisher Scientific, Loures, Portugal) and 10μg of each extract was loaded in a 12% SDS-PAGE gel. The proteins were subsequently transferred to a nitrocellulose membrane and then overlaid with 25pmol/mL of purified PPP1C gamma 1 isoform (PPP1CC1) for 1hr. Membranes were incubated with either mouse anti-His monoclonal (1:1000, Novagen, Madison, Wisconsin, USA) or rabbit CBC3C (anti-PPP1CC, 1:1000) antibodies, followed by the respective anti-mouse and anti-rabbit infrared secondary antibodies (1:5000, Li-Cor Biosciences UK Ltd, Cambridge, UK). Immunoreactive bands were then developed in Odyssey infrared-imaging system and quantified using Odyssey v1.2 software (Li-Cor Biosciences UK Ltd, Cambridge, UK). The same procedure was performed for pET-TCTEX1D4 (positive control) and pET vector (negative control).

### Statistical analysis

SigmaPlot statistical package (SigmaPlot v.11, Systat Software, San Jose, California, USA) was used for statistical analysis. Data were tested for normal distribution and homogeneity of variances. Student’s *t*-test (p<0.05, alpha=0.050) was used to detect the differences between each mutation by comparison to the control, pET-TCTEX1D4.

## Results

### Analyses of TCTEX1D4 evolution

**Figure 1:**
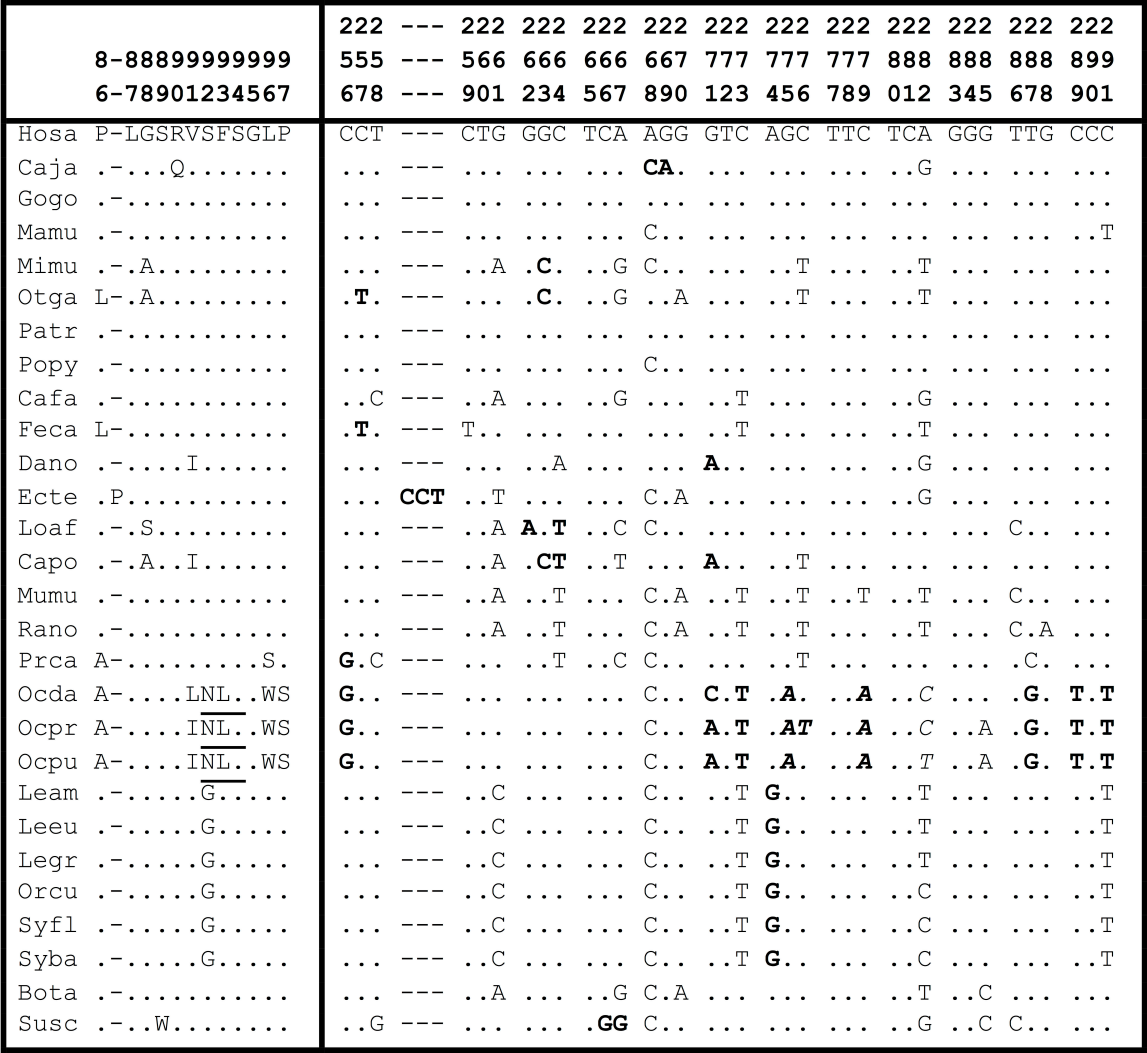
Amino acid and nucleotide sequences, corresponding to the 12 amino acids region for the 28 mammals used in this study. Bolded underlined region in the human sequence: RVSF motif; Underlined region: novel glycosylation site in Ochotona species; Bolded region: non-synonymous substitutions; Italic bolded region: nucleotide sequence corresponding to the novel glycosylation site in Ochotona species. The sequences are numbered according to human TCTEX1D4 sequence.

When comparing the TCTEX1D4 PPP1 binding motif, _90_RVSF_93_, in twenty-one mammalian species we observed that it was present in all except in *Ochotona princeps*, for which an extra glycosylation site (motif _90_NLS_92_) appears. To confirm if this was not an artifact of the database, *Ochotona dauurica* and *Ochotona pusilla* TCTEX1D4 were sequenced. Additionally, for five other lagomorph species (*Lepus* and *Sylvilagus* genera), the TCTEX1D4 coding region was partially sequenced. These sequences were compared with other mammalian sequences and translated into amino acids (Figure 1). The nucleotide substitution in *Ochotona* species generated amino acid changes that confirmed the elimination of the consensus PPP1 binding motif, _90_RVSF_93_, and the appearance of a glycosylation site. *Lepus* and *Sylvilagus* genera maintained the canonical PPP1 binding motif.

**Figure 2:**
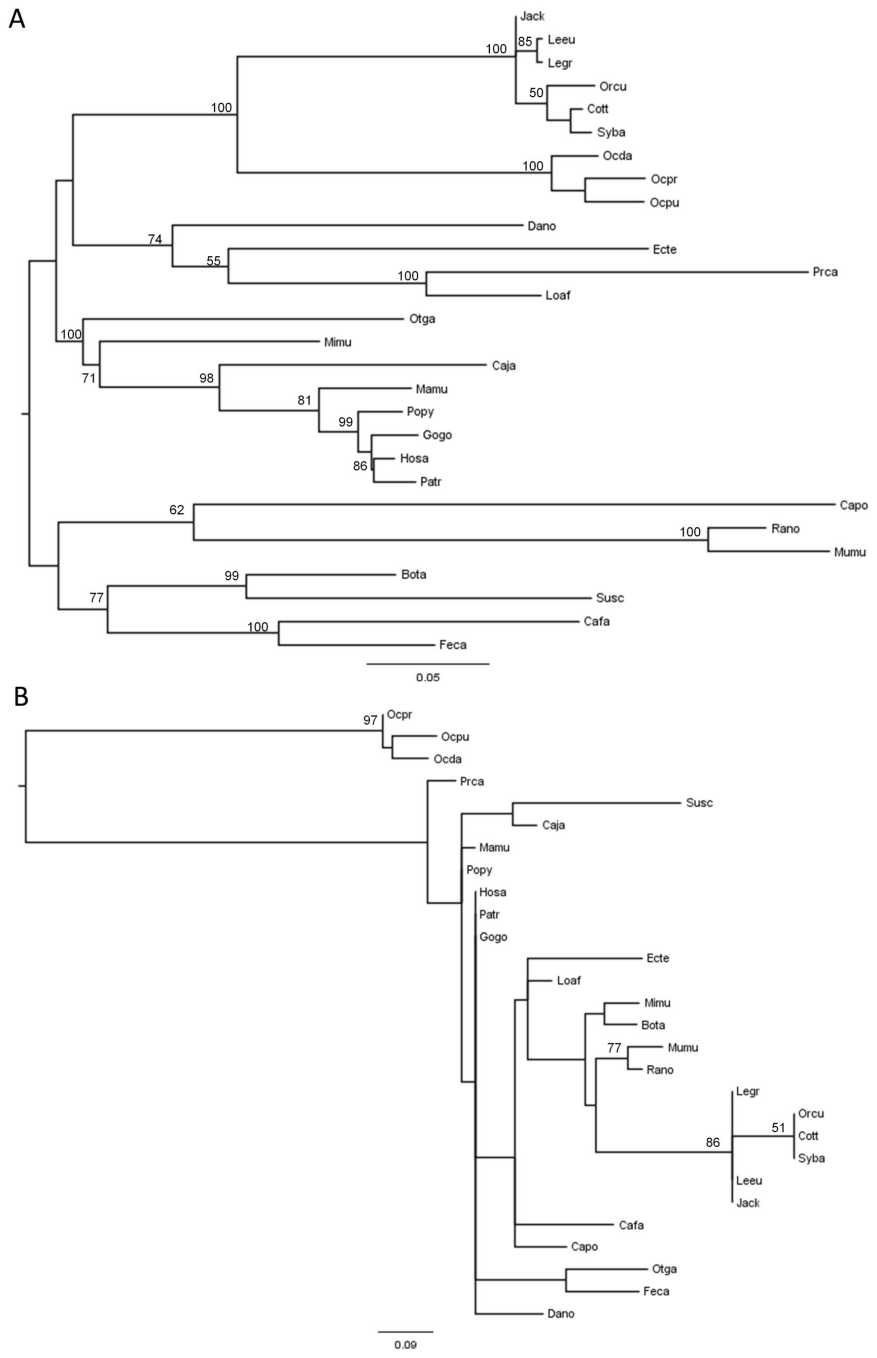
Phylogenetic reconstruction of mammalian TCTEX1D4. **A**) Maximum Likelihood tree corresponding to the whole coding region of TCTEX1D4 of the 28 mammalian species, and **B**) using only the 12-amino acid region, four upstream and four downstream of the motif _90_RVSF_93_. The analyses were performed with 1,000,000 generations and 1,000 bootstrap searches. Midpoint rooting was applied to both trees and bootstrap values > 50 are indicated on the branches.

The alignment between the sequences acquired in this work and the twenty-one sequences available in the databases for the different mammals allowed the construction of a Maximum Likelihood phylogenetic tree (Figure 2A). The topology obtained was in accordance with the mammalian taxonomy proposed and currently accepted [53], suggesting that TCTEX1D4 has been evolving under neutral selection. A new Maximum Likelihood tree (Figure 2B) was constructed using only a twelve amino acid region, corresponding to four amino acids upstream and four amino acids downstream of the motif _90_RVSF_93_. This choice of amino acids was related with PPP1 binding motif being flanked by an unusual palindromic sequence, _86_PLGS_89_, according to *Homo sapiens* sequence. As expected, the obtained tree revealed that the three *Ochotona* species formed an independent cluster, highly supported by a bootstrap value of 97 (Figure 2B).

### Pikas TCTEX1D4 - positive selection of the palindromic region

The non-synonymous to synonymous substitution ratio was calculated for the previously referred twelve amino acids. Comparing the ratios between all analyzed mammals, but excluding the three *Ochotona* species, the presented values were on average lower than 0.3, suggesting a strong purifying selection. However, when comparing ratios between the three *Ochotona* species and each of the mammalian sequences, on average, the obtained value was 1.6, suggesting that for *Ochotona* species this fragment lost the constrains on protein mutations imposed by purifying selection or/and evolved under Darwinian or positive selection. When focusing the analysis on the Superorder Glires (Order Rodentia and Order Lagomorpha), the main representatives of rodents (mouse and rat) showed a ratio of zero, meaning that TCTEX1D4 was under purifying selection for this group. When comparing rodent`s nucleotide sequences, corresponding to the twelve amino acids region, with the one from human, a total of ten substitutions causing no amino acid changes was observed (Figure 1). On the other hand, for the *Ochotona* species, a total of twelve substitutions caused six amino acid alterations. Furthermore, when comparing all species from the three Leporidae genera, *Lepus*, *Oryctolagus* and *Sylvilagus*, with the three *Ochotona* species, the ratio ranged between 1.7 and 7.0.

**Figure 3:**
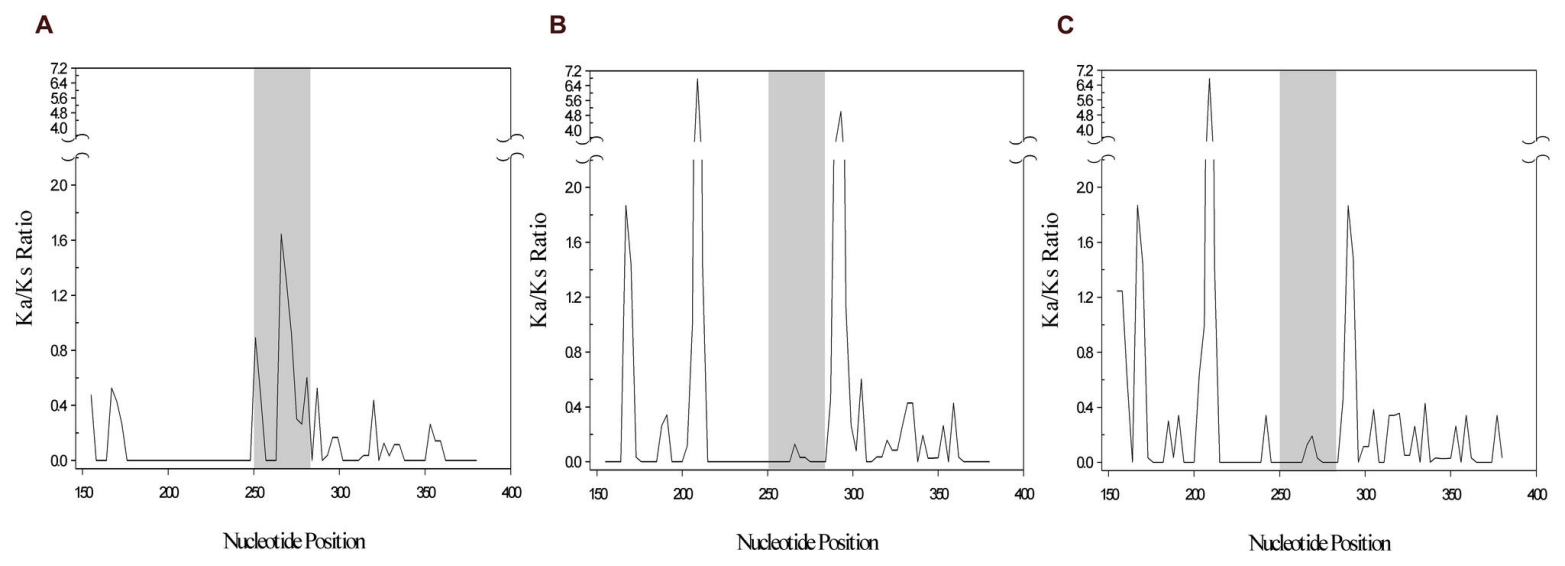
Sliding-window analysis of TCTEX1D4 palindromic region. Sliding-window analysis was performed using the DnaSP program. The non-synonymous to synonymous substitution ratio (Ka/Ks) was plotted for A) *Oryctolagus*/*Ochotona*, B) *Oryctolagus*/*Mus* and C) *Oryctolagus*/*Rattus*. Grey shaded region in the middle of the sliding windows represents the 12 amino acids palindromic region (between nucleotides 250 and 285).

These observations were visually reinforced by the sliding-window analysis of the TCTEX1D4 region, up- and downstream of the palindrome (234 nucleotides, positions 151 to 384 according to *Ochotona princeps*). When comparing *Oryctolagus* and *Ochotona* genera, the plot clearly shows a peak of positive selection in the palindromic region (position 250 to 285, Figure 3A). However, comparing *Oryctolagus* with rodents no peaks were observed in the palindromic region, which indicate that this region is under purifying selection, as in other mammals (Figure 3B & 3C). 

### TCTEX1D4 RVSF-palindrome studies

**Figure 4:**
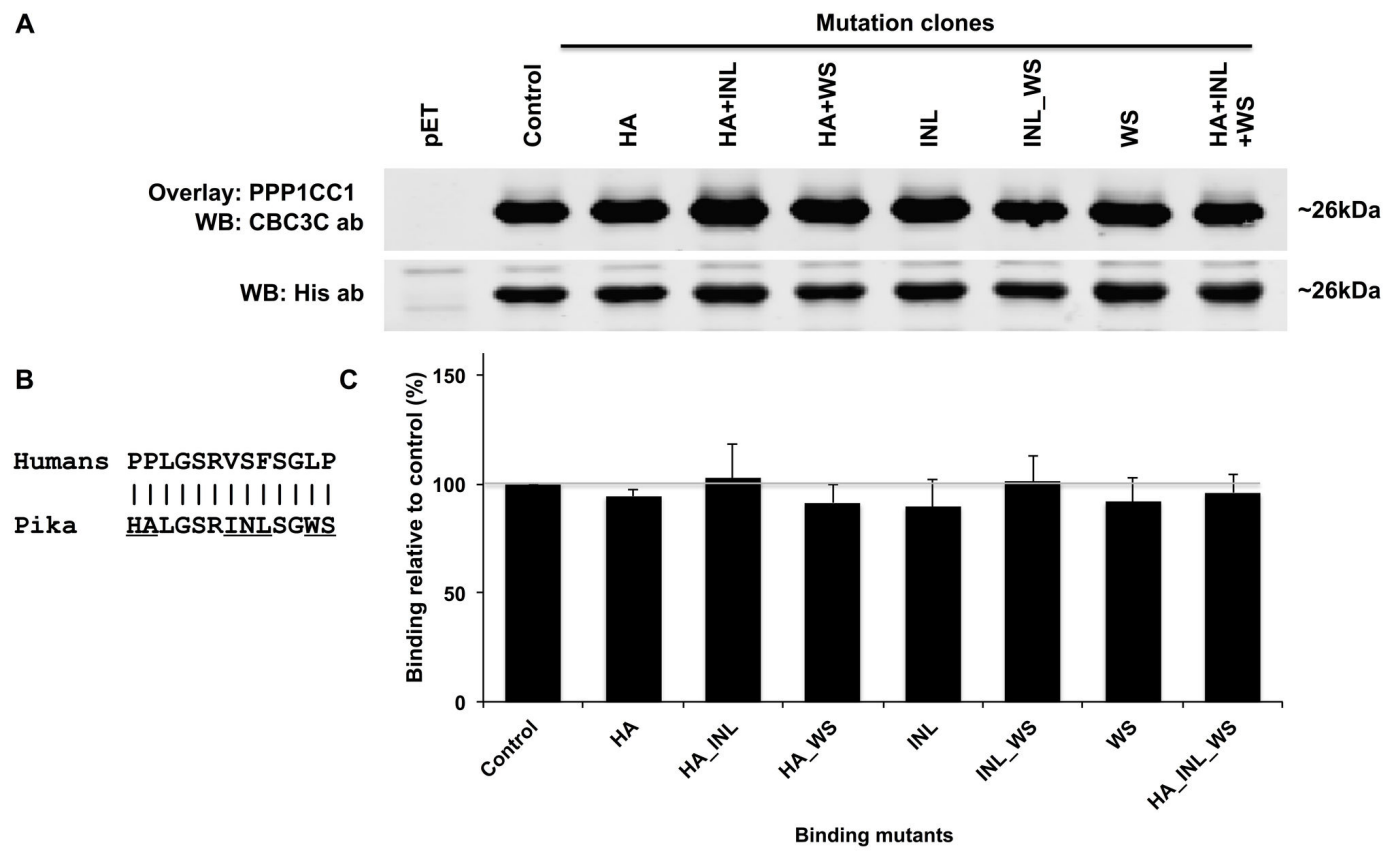
TCTEX1D4 PPP1 binding motif mutants and PPP1C binding analysis. A) Bacterial cell cultures expressing each construct were loaded in a SDS-PAGE gel (10μg). Membranes were then overlaid with purified PPP1CC and detected using CBC3C antibody. Total amount of recombinant protein was accessed using an anti-His antibody. B) Alignment of Human and Pika TCTEX1D4 PPP1 binding motif and surrounding palindromic sequences. Mutations are underlined. C) Subsequent analysis of band intensities versus total amount of recombinant protein was performed and the results plotted in a graph by comparison with the pET-TCTEX1D4 control. A Rosetta cell extract expressing pET vector alone was used as negative control. Error bars represent the standard error of the mean of triplicates. WB, Western blot.

To further study the significance of the bioinformatic studies, mutants based on the *Ochotona princeps* sequence (_83_ *HALGS* RINL *SGWS*_95_) corresponding to the human TCTEX1D4 PPP1 binding motif and flanking regions (_85_ *PPLGS* RVSF *SGLP*_97_) were generated by site-directed mutagenesis followed by bacterial expression of those mutants and PPP1C binding screening by overlay (Figure 4A & 4B). The band intensities indicate the amount of PPP1CC1 that is bound to the bacterial expressed TCTEX1D4 recombinant mutant proteins. Since all proteins were expressed with a N-terminal His-tag, anti-His antibody was used to normalize the amount of recombinant protein loaded in each lane. Subsequently band intensities were compared to the pET-TCTEX1D4 control. A Rosetta cell extract expressing pET vector alone was used as negative control.

Results show that HA+INL+WS mutant has a binding profile similar to the wild type human TCTEX1D4 since there was no statistical difference in the binding capacity. The results of the other mutations, single or double, also show no statistical difference when comparing to the control, pET-TCTEX1D4 (Figure 4C).

## Discussion

TCTEX1D4 has already been described as a new PPP1 interacting protein [22]. This new interaction was supported by the yeast two-hybrid approach, co-immunoprecipitation and overlay techniques. Previous results showed that the TCTEX1D4 N-terminal domain, where the PPP1 binding motif is present, is essential for the binding. Furthermore, *in vitro* studies with TCTEX1D4 PPP1 binding mutants strengthens the importance of the PPP1 binding motif to TCTEX1D4/PPP1C interaction [22]. Indeed, the mutation of the motif RVSF to AAAA decreases binding by 35% [22], which is surprising since the mutation of the PPP1 binding motif either to AAxA [54], RAxA [8,55] or to RVxA [56] usually abrogates PPP1 complex interaction. Nevertheless, some cases exist where interaction still occurs but to a lesser extent [57-60]. Also, there are some interacting proteins that still bind PPP1C in the presence of an excess of a synthetic RVxF peptide [59] that usually disrupts the PPP1 complex [12,61]. Other motifs besides RVxF present in these proteins and also important for the binding may at least partly explain these observations. The sequence surrounding TCTEX1D4 RVSF motif is unusual in that it contains a palindrome – *PLGS* RVSF *SGLP*. The PPP1 binding motif binds to PPP1 in a hydrophobic pocket [62]. This palindrome may form a structured arm forcing the RVSF motif, even when it is mutated into AAAA, to enter the PPP1 pocket, since the palindrome contains rigid prolines. Perhaps, if the RVSF is completely removed and the arm destroyed, TCTEX1D4 will no longer bind PPP1C.

When the twelve amino acids Maximum Likelihood tree was constructed, the three *Ochotona* species formed an independent cluster completely apart from all the other mammals. This observation shows that this fragment is unique for Pika, being the palindrome and the RVSF motif highly conserved among mammals but completely lost in Pika sequences (Figure 2 and Figure 1). This could be explained by two different hypotheses: the new motif present in the *Ochotona* species resulted from gene conversion with adjacent genes or a pattern of nucleotide substitution in this specific motif happened. Gene conversion has been reported in other mammalian genes. For example, in leporids a gene conversion event was observed between the two chromosomally adjacent genes CCR2 and CCR5, where the sequence motif _194_QTLKMT_199_ of the CCR5 protein was replaced by the HTIMRN motif, which is characteristic of CCR2 [43,63]. In the present study, none of the genes chromosomally adjacent showed a clear evidence of gene conversion with TCTEX1D4, being this event an unlikely hypothesis. Furthermore, no significant BLAST was obtained when this fragment was compared with mammalian NCBI database.

The d_N_/d_S_ ratios for the twelve amino acids region between the three *Ochotona* species and each of the mammalian sequences, is on average 1.6. When restricting the comparison to the three Leporidae genera, the ratio is further increased, for values that varied between 1.7 and 7.0. Furthermore, comparing the rodents’ nucleotide sequences corresponding to the twelve amino acids region with the one from human, a total of ten substitutions caused no amino acid changes, while for the *Ochotona* species, a total of twelve substitutions caused six amino acid alterations (Figure 1). These results were visually reinforced by the sliding-window analysis that clearly showed the palindromic region under positive selection in Pika when compared with the rest of the mammals (Figure 3). The obtained d_N_/d_S_ ratio, clearly higher than 1, and the fact that amino acid alterations created a new putative glycosylation site, highly support the hypothesis that for *Ochotona* sp. this sequence fragment has been evolving under positive selection. The occurrence of this nucleotide pattern in the three *Ochotona* species studied in this work and its absence in the other lagomorphs, suggests that this evolutionary event happened before the radiation of the *Ochotona* genus (between 6 and 20 mya) [39] and after the split of Ochotonidae and Leporidae families (between 31 and 65 mya) [36-38].

The creation of a novel putative N-glycosylation site (_90_NLS_92_) [64,65] in the three *Ochotona* species by positive selection suggests a physiologically important function. The likelihood of Pikas` TCTEX1D4 being glycosylated is increased by the fact that this motif is located more than sixty amino acids upstream of the C-terminal [66]. The remaining unsolved question is the acquired function of TCTEX1D4 in *Ochotona* species. This new putative glycosylation site may increase the half-life of the protein, which in turn will stay longer in the membrane attached to endoglin [15] being a stronger inhibitor of TGFβ in *Ochotona* sp. than in other mammals.

Furthermore, TCTEX1D4 in *Ochotona* sp. lost the PPP1 binding motif and the palindromic sequence, PLGS, probably important for the binding of TCTEX1D4 to PPP1. Thus, it would be expected that it would no longer bind to PPP1 directly. Evolutionarily, it is not clear what happened first, if the loss of the palindrome with subsequent mutation of the PPP1 binding motif to a glycosylation site or the acquisition of a glycosylation site by positive selection followed by loss of the palindrome. These evolutionary analyses suggest that an alternative mechanism may exist in *Ochotona* for TCTEX1D4 binding to PPP1. Thus, we employed an overlay screening with different binding mutants to test this hypothesis. Results show that Pika TCTEX1D4 aberrant RVxF motif and respective non-palindromic surrounding region, _83_ *HALGS* RINL *SGWS*_95_, sustain the binding of the TCTEX1D4 mutant to PPP1CC, at the same levels of the wild type human TCTEX1D4 (Figure 4). Moreover, in single and double mutants, the binding capacity was also maintained, which clearly shows that although substantial differences were found in Pikas RVxF and surrounding regions, these do not contribute to the disruption of the binding. Earlier results have shown that a mutation of the RVSF motif to AAAA only decreases the overall binding efficiency in 35% [22]. Furthermore, we have also shown that important regions for this binding are concentrated in the N-terminal, where the RVxF is also present. This means that, either the RVxF motif is not the only point of contact, or the RVxF surrounding region is also important for this binding. Here, using Pika aberrant motif we clearly show that the second hypothesis does not explain why the binding is not abolished when we mutate the RVxF motif.

PPP1 binding motif RVxF motif is usually surrounded by basic residues (arginine, lysine and histidine) in the N-terminal and by acidic residues (aspartate and glutamate) in the C-terminal [8,25]. Analysis of 143 RVxF motifs in known and novel PPP1 interacting proteins revealed that five to six of these flanking basic and acidic residues are relatively common among PPP1 interacting proteins [3]. Human TCTEX1D4 RVSF motif is a strong motif according to this analysis but the palindromic region that surrounds it does not follow this pattern, since no basic or acidic amino acids are present. Even so, all the flanking residues are present at some extent in other PPP1 interacting proteins. By comparing the above results with ours, the PP to HA mutation would not lead to any difference because some PPP1 interacting proteins also have these amino acids in these positions (P 11%, P 4% comparing to H 4%, A 10%). Regarding the VSF to INL mutation (V 94%, S 21%, F 83% comparing to I 6%, N 5% and L 0%) we can infer that the binding would be potentially abrogated, but our results show that it is maintained. Finally, relatively to the LP to WS mutation (L 3%, P 7% comparing to W 0%, S 7%) our results show that this mutation does not alter the binding. Taken together, our results show that the palindromic sequence, evolutionarily conserved, appears to be irrelevant for the binding, since the HA and WS mutations resulted in the same binding capacity, and that the unique RVxF motif, RINL, seems to sustain the binding. The results show undoubtedly that even with the motif and flanking regions evolving under positive selection, both regions seem to still sustain the binding capacity. The hypothesis of another N-terminal region important for the binding arises and might suggest that the RVxF motif is just a point of contact that helps to stabilize the complex.

In conclusion, TCTEX1D4 evolutionary analysis revealed that in Pika the PPP1 binding motif was lost and replaced by a new putative glycosylation site. Additionally, we also observed, in *Ochotona*, the loss of a highly conserved palindrome present among mammals. The presence of the HA, INL and WS substitutions in *Ochotona*, does not alter the binding capacity. The combination of these factors in Pika species makes these a perfect model to study the biology of PPP1/TCTEX1D4 complex and can be expanded to understand PPP1 complexes, increasing the number of interacting proteins previously expected to exist based on the consensus RVxF motif.

## Supporting Information

Table S1 **List of mammalian species used in this study in which the coding sequence of TCTEX1D4 was retrieved from NCBI or ENSEMBL.**
(DOCX)
