# Supplementary material for: An Intriguing Shift Occurs in the Novel Protein Phosphatase 1 Binding Partner, TCTEX1D4: Evidence of Positive Selection in a Pika Model
Source: PLoS One. 2013 Oct 10;8(10):e77236. doi: 10.1371/journal.pone.0077236 (PMC3795061; doi:10.1371/journal.pone.0077236)
Supplement: Table S1 — List of mammalian species used in this study in which the coding sequence of TCTEX1D4 was retrieved from NCBI or ENSEMBL. (DOCX) [file pone.0077236.s001.docx]

Supplementary table 1. *List of the mammalian species in which the coding sequence of TCTEX1D4 was retrieved from NCBI or ENSEMBL and used in this study.*

| **Species name** | **Common name** | **Database ID** | **Database Source** |
| --- | --- | --- | --- |
| *Bos taurus* (Bota) | Cow | XM_594476 | NCBI GenBank |
| *Callithrix jacchus* (Caja) | [Marmose](http://www.ensembl.org/Callithrix_jacchus/Info/Index)t | Chromosome 7: 79,571,655-79,572,320 | Ensembl |
| *Canis familiaris* (Cafa) | Dog | Chromosome 15: 18,523,787-18,524,446 | Ensembl |
| *Cavia porcellus* (Capo) | [Guinea Pig](http://www.ensembl.org/Cavia_porcellus/Info/Index) | scaffold_165: 1,051,684-1,052,355 | Ensembl |
| [*Dasypus novemcinctus*](http://www.ensembl.org/Homo_sapiens/Search/Details?_C=eJwFwWEKgCAMBtDvKOEFTCiIDtAF6r!ILgqGszGj4*deZFBFfCsKnamzIT6wbPSFMg1ZtIkmI29KtLpDmt!layZP8!IQr9u2zoww4genQxhA&_c=%2b13839964057159149480) (Dano) | Armadillo | GeneScaffold_5913: 7,015-7,671 | Ensembl |
| *Echinops telfairi* (Ecte) | [Lesser Hedgehog Tenrec](http://www.ensembl.org/Echinops_telfairi/Info/Index) | GeneScaffold_7046: 35,805-36,470 | Ensembl |
| *Felis catus* (Feca) | Cat | GeneScaffold_2486: 79,592-80,251 | Ensembl |
| *Gorilla gorilla* (Gogo) | Gorilla | Chromosome 1: 46,433,826-46,434,515 | Ensembl |
| *Homo sapiens* (Hosa) | Human | NM_001013632 | NCBI GenBank |
| *Loxodonta africana* (Loaf) | Elephant | SuperContig scaffold_34: 24,862,938-24,863,606 | Ensembl |
| *Macaca mulatta* (Mamu) | Rhesus Monkey | XM_001099595 | NCBI GenBank |
| *Microcebus murinus* (Mimu) | Mouse Lemur | GeneScaffold_1367: 59,896-60,555 | Ensembl |
| *Mus musculus* (Mumu) | Mouse | NM_175030 | NCBI GenBank |
| *Ochotona princeps* (Ocpr) | American Pika | GeneScaffold_4323: 138,892-139,548 | Ensembl |
| *Oryctolagus cuniculus* (Orcu) | European Rabbit | scaffold_0: 101,766,508-101,767,167 | Ensembl |
| *Otolemur garnetti* (Otga) | Bushbaby | GeneScaffold_2671: 80,377-81,036 | Ensembl |
| *Pan troglodytes* (Patr) | Chimpanzee | Chromosome 1: 45,543,578-45,544,952 | Ensembl |
| *Pongo pygmaeus* (Popy) | [Orangutan](http://www.ensembl.org/Pongo_pygmaeus/Info/Index) | Chromosome 1: 185,125,020-185,125,685 | Ensembl |
| *Procavia capensis* (Prca) | Hyrax | GeneScaffold_6128: 11,550-12,212 | Ensembl |
| *Rattus norvegicus* (Rano) | Rat | XM_233427 | NCBI GenBank |
| *Sus scrofa* (Susc) | Pig | NM_001032356 | NCBI GenBank |
